# Aerobic Photobiocatalysis Enabled by Combining Core–Shell Nanophotoreactors and Native Enzymes

**DOI:** 10.1021/jacs.2c00576

**Published:** 2022-04-01

**Authors:** Wenxin Wei, Francesca Mazzotta, Ingo Lieberwirth, Katharina Landfester, Calum T. J. Ferguson, Kai A. I. Zhang

**Affiliations:** ‡ Max Planck institute for Polymer Research, Ackermannweg 10, 55128 Mainz, Germany; $ Department of Materials Science, Fudan University, 200433 Shanghai, People’s Republic of China

## Abstract

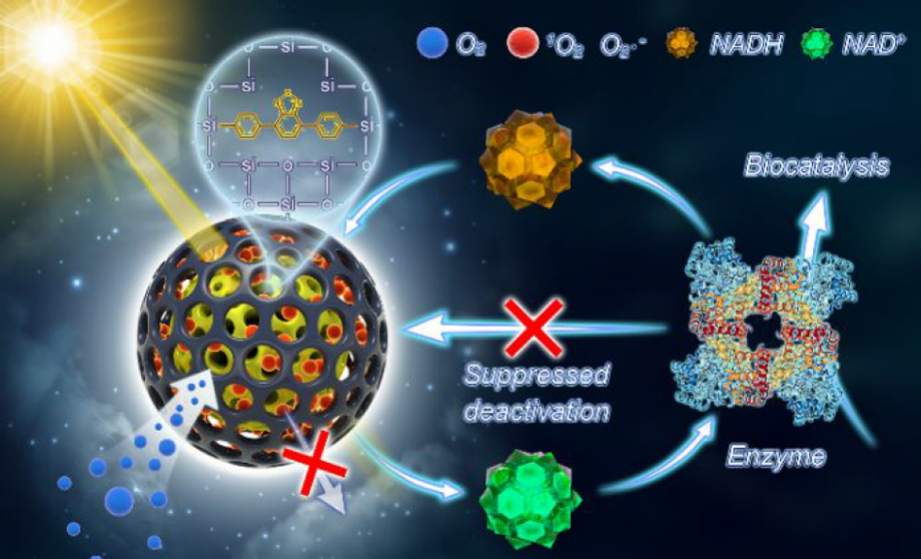

Biocatalysis has become a powerful tool in synthetic chemistry, where enzymes are used to produce highly selective products under mild conditions. Using photocatalytically regenerated cofactors in synergistic combination with enzymes in a cascade fashion offers an efficient synthetic route to produce specific compounds. However, the combination of enzymes and photocatalysts has been limited due to the rapid degradation of the biomaterials by photogenerated reactive oxygen species, which denature and deactivate the enzymatic material. Here, we design core–shell structured porous nano-photoreactors for highly stable and recyclable photobiocatalysis under aerobic conditions. The enzymatic cofactor NAD^+^ from NADH can be efficiently regenerated by the photoactive organosilica core, while photogenerated active oxygen species are trapped and deactivated through the non-photoactive shell, protecting the enzymatic material. The versatility of these photocatalytic core–shell nanoreactors was demonstrated in tandem with two different enzymatic systems, glycerol dehydrogenase and glucose 1-dehydrogenase, where long-term enzyme stability was observed for the core–shell photocatalytic system.

## Introduction

Biocatalysis has recently emerged as a considerably competitive tool in synthetic organic chemistry.^1−5^ Enzymatic systems have been implemented to produce highly selective compounds under milder conditions, where enzymatic systems often require fewer synthetic steps and lower reaction temperatures and use greener solvents, compared to traditional synthetic methods.^6,7^ Moreover, in nature, enzymes have developed to mediate a myriad of different reactions, resulting in a vast array of potential high-value products. To date, a broad range of enzyme classes have been employed including halogenases,^8−10^ oxidoreductases,^11−19^ and hydrolytic enzymes.^20−22^ Some enzymes, such as dehydrogenases, are cofactor-dependent enzymes, limiting their broad industrial application.^23^ Cofactors such as nicotinamide cofactors NADH (b-nicotinamide adenine dinucleotide) or NADPH (b-nicotinamide adenine dinucleotide phosphate) in their reduced forms are typically used by oxidoreductases in order to reduce a specific substrate. In nature complicated electron transport chains exist in order to regenerate the required reduced cofactor species.^24^ However, the synthetic replication of these natural systems has proven to be extremely challenging and rather difficult to maintain; therefore, alternative cofactor regeneration pathways have been investigated.

Visible-light-mediated photocatalysis has been developed as a way to catalyze reactions under mild conditions. Typically, either photocatalysts based on transition metals or organic catalysts have been investigated.^15,25−27^ Recently, the implementation of photocatalytic systems for cofactor regeneration has been investigated. To date, many photocatalysts, such as TiO_2,_^14^ organic dyes,^28^ carbon quantum dots,^29^ graphitic carbon nitride (g-C_3_N_4_),^19,30−34^ covalent organic frameworks (COFs),^35−37^ metal organic frameworks (MOFs),^35,38,39^ conjugated microporous polymers,^40^ and classical photocatalytic polymers,^41^ have shown great potential to regenerate enzymatic cofactors such as NADH/NAD^+^.^42,43^ Unfortunately, these photocatalytic systems also produce active oxygen species (ROS), which currently limits the combination of these materials with enzymes, due to rapid degradation of the biomaterial. There is therefore an urgent need to develop new photocatalytic materials with the ability to prevent the deactivation and degradation of biomaterials.

To mitigate the rapid degradation of enzymes, tandem photocatalyst–enzyme systems have been introduced, where reactions are often undertaken in anaerobic conditions, removing oxygen from the system.^44^ Indeed, this does prevent enzyme degradation by photocatalytic byproducts, but it can significantly reduce the efficiency of the enzymatic and photocatalytic materials. Alternatively, the immobilization of the enzymatic material within silica nanoreactors has been investigated to eliminate contact of ROS from the biomaterial.^45^ Furthermore, superoxide dismutase can be coencapsulated to further protect the active enzymatic material.^46^ However, these methods require the immobilization of the enzymatic material within a solid construct, which can significantly decrease its efficiency compared to the native enzyme.

Ideally, the synergistic combination between photocatalysts and enzymes in their native state would be achieved. Here, photocatalytic materials should be readily dispersible in aqueous environments and be able to regenerate enzymatic cofactors, while not effecting the efficiency of the enzymatic system. To achieve this, photocatalytic materials should be designed so that they possess hydrophilicity and enable easy portioning of the cofactors into an active center, where they can be rapidly converted. As for morphology, well-designed mesoporous structures can be further endowed with high specific surface area and accessible active sites compared to bulk or materials with smaller pores, further facilitating mass transfer and reaction efficiency.^47,48^ Contrastingly, the photocatalytic material should not allow generated active oxygen species to come in contact with the required biomaterials. To achieve this, the relatively short lifetime of ROS can be exploited. The photocatalytic material can be engineered so that the diffusion of ROS from within the photocatalytic material exceeds the lifetime of ROS.

Here, we have designed core–shell structured nano-photoreactors, where the photocatalytic unit is concentrated in the particle core and surrounded by a mesoporous silica outer shell. This configuration allows the enzymatic cofactors to easily diffuse through the porous shell to the photoactive center, where they can be rapidly transformed to their active state. Conversely, the shell is produced so that, by size exclusion, enzymes can be physically separated from the active photocatalytic core. The mesoporous shell of the photocatalyst is designed so that the diffusion time of the active oxygen, out of the photocatalytic material, is long enough that the active oxygen species return to the harmless ground state to the biomaterials. This core–shell photocatalyst design, in combination with an enzyme species, enables the continuous production of a desired product under visible light irradiation. The photobiocatalytic activity was tested in tandem with two different enzymatic systems, glucose dehydrogenase and glycerol dehydrogenase, where long-term enzyme stability was observed for the core–shell nano-photoreactor.

## Results and Discussion

**Figure 1:**
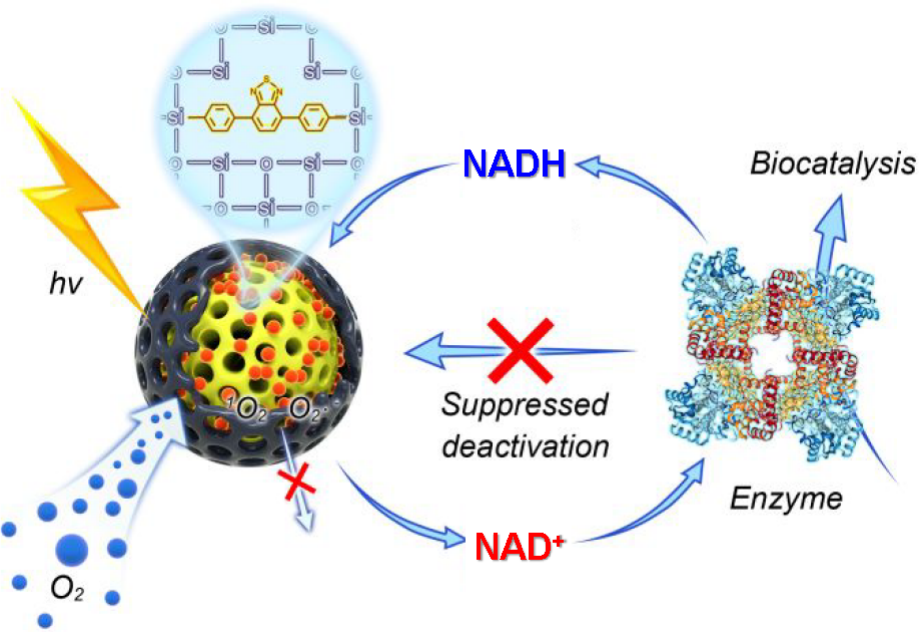
Enzyme and photocatalyst combination system. The mesoporous core–shell nano-photoreactor completes the NADH/NAD^+^ cycle together with the enzyme and promotes the enzyme to catalyze the bioreaction in cascade while preventing enzyme deactivations by active oxygen species.

The tandem system of an enzyme and the core–shell nano-photoreactor is depicted in Figure 1. The photocatalytic core material is formed from a diphenylbenzothiadiazole (BTPh_2_) unit modified with two organosilane groups and tetraethoxysilane (TEOS), producing a porous photocatalytic nanoparticle (NP-C). This active core is encased in a secondary mesoporous silica layer to form a core–shell structure (NP-CS).^49^ The synthesis and experimental details are described in the Supporting Information (SI). The core–shell nanoparticles allow the diffusion of NADH into the particle, where it is oxidized to NAD^+^ under photocatalytic conditions. Concurrently, oxygen is converted to active oxygen species such as singlet oxygen, superoxide, etc., but they are then “trapped” within the highly porous shell layer, where they return to the ground state due to their limited lifetimes, becoming harmless to the biomaterials. The enzyme reduces the NAD^+^ generated by the photocatalyst to NADH, where this electron transfer is used to undertake the desired chemical transformation. To demonstrate the versatility of this new photobiocatalytic system under aerobic conditions, two different enzymatic systems were tested over multiple cycles.

**Figure 2:**
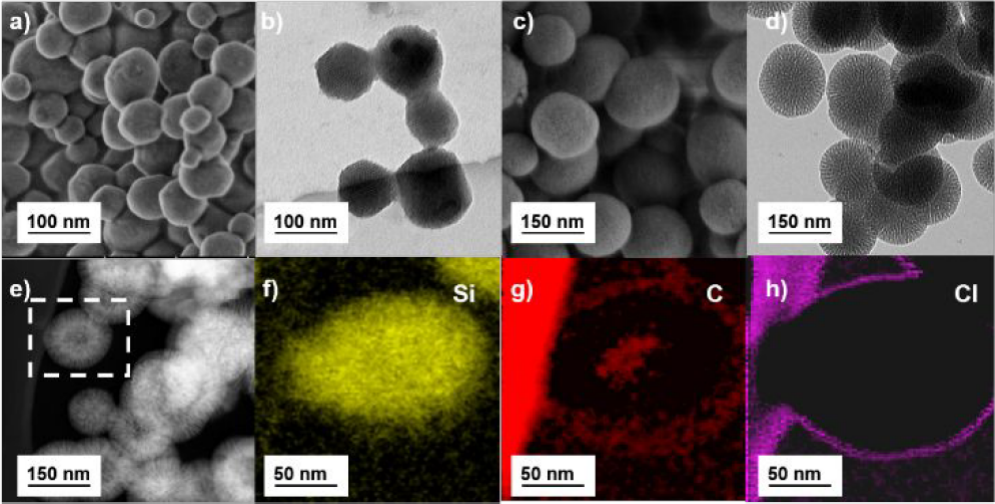
SEM and TEM image of the (a and b) photocatalytic core (NP-C) and (c and d) core–shell (NP-CS). (e) Annular dark-field (ADF) image showing the selected nanoparticle for (f)–(h) elemental mapping, which shows enriched contents of silicon in the shell and carbon in the core.

To investigate the formation of the nanoparticles, transmission electron microscopy (TEM) and scanning electron microscopy (SEM) were conducted. As shown in Figure 2, the SEM and TEM images show that the synthesized nanoparticles have a porous structure. The core of the photocatalytic material has a size range of 80–100 nm, while the core–shell nanoparticles have a size of around 130 nm. The hydrodynamic radius of the photocatalytic core and the shell was investigated, and a similar increase in size was observed from 200 to 250 nm (Figure S1).

Energy dispersive X-ray (EDX) spectroscopy mapping was used to determine the composition of the photocatalytic particles. Elemental mapping was undertaken to determine the morphology of the core–shell particles. From the annular dark-field image we can see the nanoparticle selected Figure 2e. Here, a difference in contrast between the core and the shell of the particle is observed, suggesting a difference in the materials’ composition in these two regions. Electron energy loss spectroscopy (EELS) was used to determine the elemental composition of the nanoparticles. Silicon was detected throughout the particle, as expected due to the use of TEOS in both the core and the shell (Figure 2f). Carbon was detected on the surface of the nanoparticle (again due to the surfactant) and within the core. The carbon-rich core is due to the presence of the photocatalytic aromatic BTPh_2_ moiety, showing that it was successfully incorporated into the core particle (Figure 2g). A strong signal for chlorine was detected on the shell of the nanoparticles (Figure 2h); this originates from the surfactant cetyltrimethylammonium chloride (CTAC) that was used in the formation of the nanoparticles as stabilizer. From the carbon elemental cross-section (Figure S2), we can see a clear carbon-free area corresponding to the shell of around 25 nm either side of the core. This would suggest that this region is free of the photocatalyst and is pure silica, supporting the core–shell morphology of the nanoparticles.

**Figure 3:**
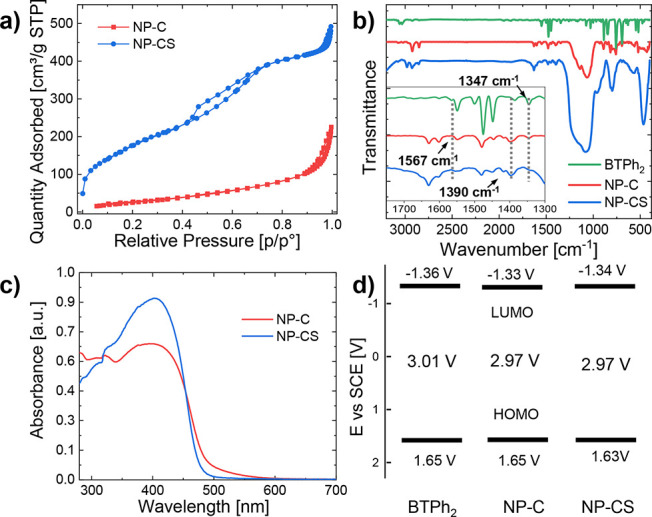
(a) Gas sorption isotherms, (b) Fourier transform infrared (FTIR) spectra, (c) diffuse reflectance UV/vis spectra, and (d) HOMO/LUMO band positions of small molecular BTPh_2_ and photocatalytic nanoparticles NP-C and NP-CS.

To characterize the porosity of the core and core–shell nanoparticles, nitrogen gas sorption at 77 K was used (Figure 3a). The pore size of the materials was measured by BJH (Barrett–Joyner–Halenda) pore size analysis, and the major peak of the pore size distribution for the photocatalytic core material was centered on 2.5 nm (Figure S3). Two peaks were observed for the core–shell material: one centered at 2.5 nm (corresponding to the core) and one centered at 3.9 nm (corresponding to the shell). The shell pore diameter was designed so that it is smaller than the typical enzyme diameter, separating them from the active core. The BET (Brunauer–Emmett–Teller) surface area of the photocatalytic core and core–shell were determined over a relative pressure range of 0.01–0.3 *P*/*P*_0_ (Figure S4). The surface areas of the photocatalytic core and core–shell material were found to be around 102 and 664 m^2^ g^–1^.

The Fourier transform infrared spectrum (FTIR) was obtained to show the incorporation of benzothiadiazole within the photocatalytic core. Peaks corresponding to 1347, 1390, and 1567 cm^–1^, which are characteristic for C=N and N–S stretching modes in the benzothiadiazole moiety, were observed.^50^ The strong and wide absorption signals at 1095 cm^–1^ can be assigned to a Si–O–Si antisymmetric stretching vibration^51^ (Figure 3b). Similar conclusions could be drawn from the corresponding solid-state ^13^C cross-polarization magic-angle-spinning (CP-MAS) NMR spectrum (Figure S5). The pronounced signals at ca. 153, 134, and 129 ppm verified the presence of the BTPh_2_ unit within the nano-photoreactor, and the signal at 6 ppm was considered to be the residual surfactant.^52^

UV/vis diffuse reflectance (DR) measurement was then used to characterize the optical properties of NP-C and NP-CS nanoparticles. As shown in Figure 3c, the maximum absorption for both the core and core–shell particles was at 410 nm. The absorption range of the nanoparticles extends to around 550 nm for the core particles, whereas the core–shell particles extend to around 500 nm. Currently, the cause of this difference is unknown, but both extend into the visible light spectrum. The emission range of the core particle of the photoluminescence spectrum is between 450 and 650 nm, and the maximum is 512 nm; the range of the core–shell particle is between 425 and 650 nm, and the maximum is 509 nm, which is similar to that of the BTPh_2_ unit (Figure S6).^53,54^ From the Kubelka–Munk function, it can be concluded that the optical band gaps of NP-C and NP-CS are 2.84 and 2.81 eV, respectively (Figure S7). In order to evaluate the positions of the lowest unoccupied molecular orbital (LUMO) and the highest occupied molecular orbital (HOMO) of the material in this study, cyclic voltammetry (CV) measurements were performed (Figure S8). The HOMO/LUMO of NP-C and NP-CS were determined to be 1.65 V/–1.33 and 1.63 V/–1.34 V vs a saturated calomel electrode (SCE). This corresponds to the similar levels obtained for BTPh_2_, with a HOMO/LUMO level of 1.65/–1.36 V vs SCE (Figure 3d). It demonstrates that the incorporation of the photocatalytic moiety into the silica nanoparticle does not significantly affect the electronic structure of the material.

**Figure 4:**
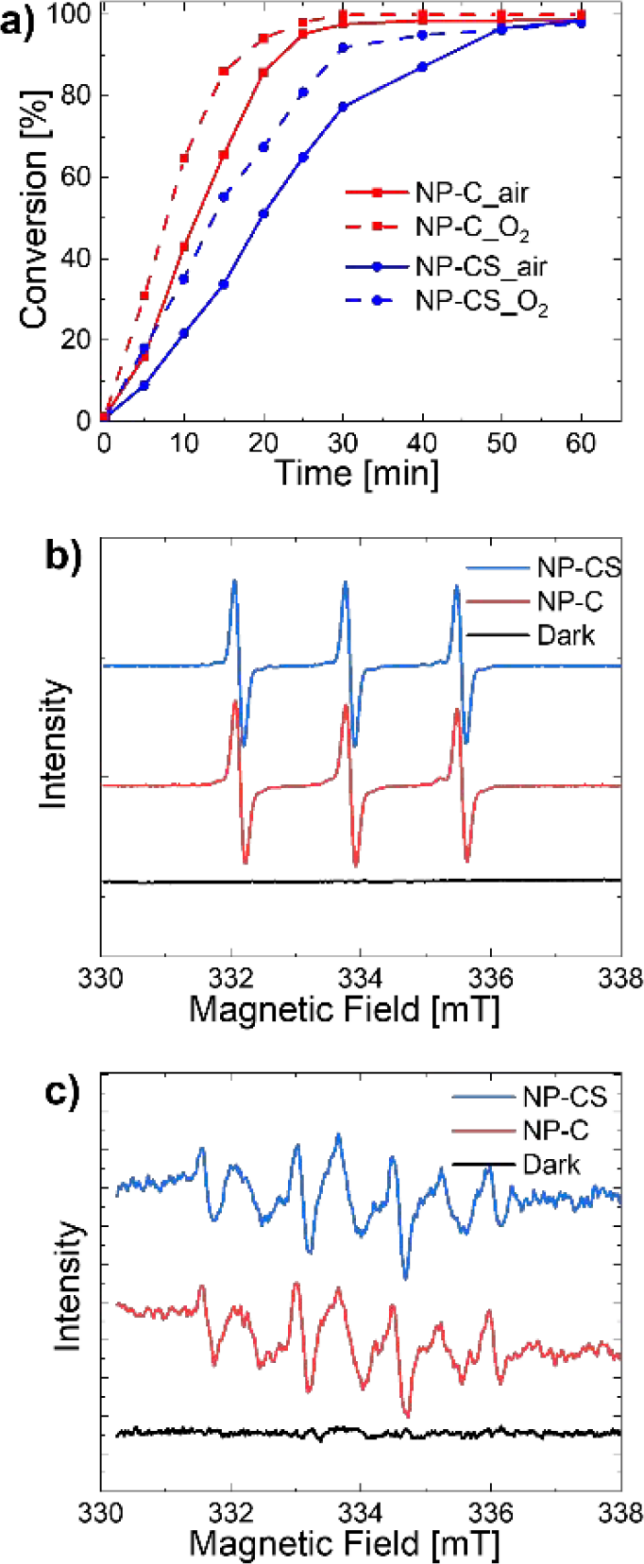
(a) Kinetic curve of NADH oxidation with NP-C and NP-CS in air and O_2_ atmosphere, respectively; EPR spectra of NP-C and NP-CS with (b) TEMP and (c) DMPO in the dark or under light irradiation.

The ability of the photocatalytic particles to oxidize NADH was investigated, comparing both the core and the core–shell particles (Figure 4a). The concentration of the core (e.g., the photoactive portion) was kept constant at 1 mg·mL, and the rate of the reaction was found to be quicker for the core particles compared to the core–shell, with 100% conversion in 30 and 50 min, respectively. This difference was attributed to the diffusion time of NADH through the shell to the catalytic core.

**Figure 5:**
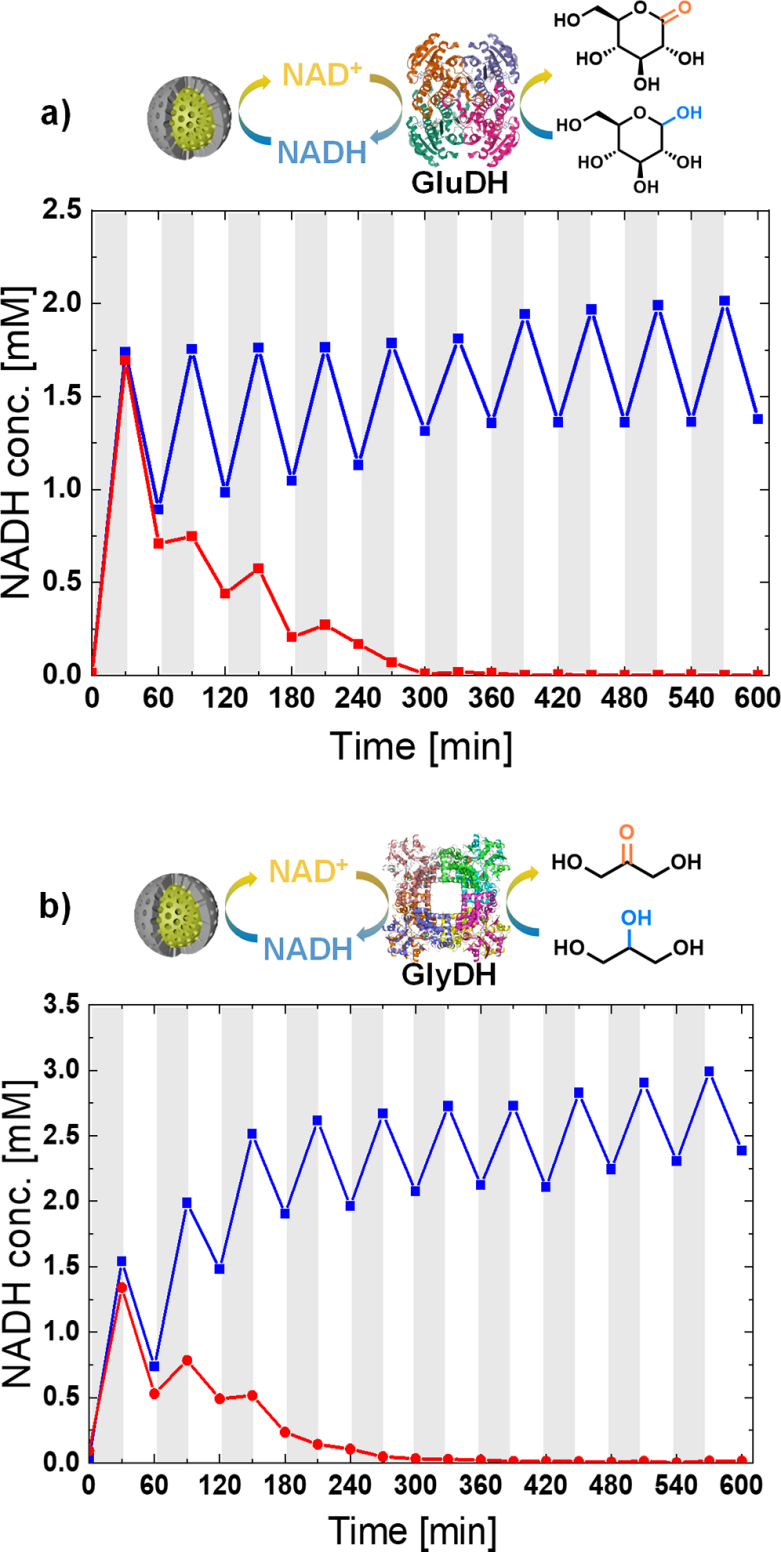
Core (red line) and core–shell (blue line) nano-photoreactor combine with the enzyme (a) glycerol dehydrogenase and (b) glucose dehydrogenase to recover NAD^+^ and NADH by alternately adding reagents (glycerol and glucose) and light in the dark.

The presence of ROS species was determined using electron paramagnetic resonance (EPR) spectroscopy by employing trapping agents 2,2,6,6-tetramethylpiperidine (TEMP) (Figure 4b) and 5,5-dimethyl-1-pyrroline-*N*-oxide (DMPO) to detect common ROS species as singlet oxygen and superoxide or hydroxide radical, respectively (Figure 4c).^55,56^ Here, the trapping agents are sufficiently small that they can diffuse into the core of the photocatalytic nanoparticle and detect the production of ROS. For both the core and the core–shell particles, singlet oxygen and superoxide were detected. It should be mentioned that besides superoxide, significant amounts of mainly hydroxyl (HO^•^) radicals could be detected in illuminated samples due to the pattern.^11^ These hydroxyl radicals most likely originate from water oxidation, from the reaction of superoxide (O_2_^•–^, from O_2_ reduction). However, due to the much larger size of enzymes, it is unlikely that they are able to diffuse in and come in contact with the active photocatalytic core.^57,58^

The light-driven regeneration of the enzymatic cofactor NAD^+^ and subsequent utilization by enzymatic material was tested using two model enzyme systems, glucose 1-dehydrogenase and glycerol dehydrogenase. Both of the enzymes chosen are oxidoreductases, a class of enzymes that has shown great promise for use in industrial processes.^18^ Here, both enzymes are dependent on NAD^+^ to oxidize a selective substrate, which can be regenerated by the photocatalytic material.

The designed photobiocatalytic system was initially tested using glucose 1-dehydrogenase, which converts glucose and NAD^+^ into gluconolactone and NADH. Here, NAD^+^ and glucose were added into the system, under dark conditions, enabling the enzyme to use the oxidized cofactor to convert glucose (Figure 5a). After 30 min, the sample was irradiated with blue LED for a further 30 min. During this period a decrease in the NADH concentration was observed, due to photocatalytic conversion back to NAD^+^. From this, we can see that the photocatalytic rate of conversion of NADH to NAD^+^ is much faster than the corresponding enzymatic reaction. After the photocatalytic regeneration of NAD^+^ again the lights were turned off, and for the system containing core–shell particles the NADH conversion rose again. This shows that the enzyme is still in its active form and has not been denatured. In contrast, only a small increase in NADH concentration was observed for the system in the dark containing the unprotected core photocatalyst. The core–shell system could be successfully cycled over multiple iterations, without significant reduction in enzymatic activity. Conversely, complete loss in enzyme activity was observed after three cycles in the unprotected system.

To demonstrate the versatility of this photocatalytic system for use in photobiocatalysis, a second enzymatic system was investigated. Glycerol dehydrogenase was used to convert glycerol and NAD^+^ to dihydroxyacetone and NADH (Figure 5b). Similarly to the system with glucose 1-dehydrogenase multiple light–dark cycles were used to demonstrate the synergistic combination of the photobiocatalytic system, with no loss in activity after 10 cycles observed. Once again, the unprotected photocatalytic system resulted in rapid degradation of the biomaterial, showing the need for the protecting mesoporous silica shell.

We also investigated the effect of NP-CS with different shell morphologies or thicknesses on photobiocatalysis. Two more samples, NP-C with a nonporous shell and NP-CS with a shell thickness of about 40 nm, were synthesized by the Stöber process and biphase stratification approach under a longer shell growth time.^49,59^ A comparison of the TEM images is shown in Figure S13. The combination of glucose 1-dehydrogenase and the photocatalyst system was chosen as control experiments. As shown in Figure S16, under the same conditions as in Figure 5, using NP-C with a nonporous shell, no NAD^+^ was generated. This indicates that likely no mass transfer from outside to photocatalyst core was possible and the reaction did not occur. The nanoreactor with a thicker shell (∼40 nm) showed a lower reaction rate. And similar to the initial photoreactor with a shell thickness of ∼25 nm, the process was stable and fully recyclable. This indicates that a thicker shell did slow down the reaction rate due to a longer diffusion time of NADH and NAD^+^.

## Conclusion

In summary, core–shell structured nano-photoreactors have been synthesized that enable stable and recyclable aerobic photobiocatalysis to be undertaken. The obtained core–shell nanoparticles are composed of a photocatalytically active core and a silica shell, which have a mesoporous structure that facilitates easy diffusion through the material. This core–shell structure protects biomaterials from the active oxygen species such as superoxide radicals and singlet oxygen produced by the photocatalyst. Aerobic photobiocatalytic cascade reactions could be undertaken, where the photoactive material regenerates the cofactor NAD^+^; this oxidized cofactor could then subsequently be used by two different oxidoreductases reducing the cofactor back to NADH. This circular process could be repeated over 10 consecutive cycles, demonstrating the stability of this system. Critically, this system keeps the enzymatic material in its native and most active state, ensuring a highly efficient conversion. So far the use of this system has been limited to model enzyme systems, but in principle any NAD^+^-dependent enzyme could be used in this system. This paves the way for industrially relevant photobiocatalytic systems to be investigated in the future.

## Supporting Information Available

Details of the synthetic route of the core–shell photoreactor, characterization methods, photobiocatalysis and control experiments, etc. (PDF)
