# Correction to “Aerobic Photobiocatalysis Enabled by Combining Core–Shell Nanophotoreactors and Native Enzymes”

**DOI:** 10.1021/jacs.3c00723

**Published:** 2023-02-01

**Authors:** Wenxin Wei, Francesca Mazzotta, Ingo Lieberwirth, Katharina Landfester, Calum T. J. Ferguson, Kai A. I. Zhang

Page 7324. The following was omitted from the Acknowledgments in the original publication.

