# Supplementary material for: Aerobic Photobiocatalysis Enabled by Combining Core–Shell Nanophotoreactors and Native Enzymes
Source: J Am Chem Soc. 2022 Apr 1;144(16):7320–6. doi: 10.1021/jacs.2c00576 (PMC9052756; doi:10.1021/jacs.2c00576)
Supplement: Supplementary file 1 — ja2c00576_si_001.pdf [file ja2c00576_si_001.pdf]

Supporting Information

Aerobic photobiocatalysis enabled by combining core-shell nano-photoreactors and native enzymes

Wenxin Wei, Francesca Mazzotta, Ingo Lieberwirth, Katharina Landfester,* Calum T. J. Ferguson* and Kai A. I. Zhang*

Materials

1-Bromo-4-iodobenzene, triethoxysilane, tetraethylorthosilicate, bromobenzene, 2,1,3-benzothiadiazole-4,7-bis(boronic acid pinacol ester), allylmagnesium bromide, hexadecyltrimethylammonium chloride, hexadecyltrimethylammonium bromide, bis(acetonitrile)(1,5-cyclooctadiene)rhodium(I)tetrafluorobotate, tetrakis(triphenylphosphine)palladium(0) were purchased from Sigma-Aldrich Chemie GmbH (Steinheim, Germany). β-nicotinamide adenine dinucleotide reduced disodium salt hydrate, β-nicotinamide adenine dinucleotide sodium salt were purchased from Carl Roth GmbH & Co. KG (Karlsruhe, Germany) and used without further purification. Glycerol dehydrogenase from Cellulomonas sp. and glucose dehydrogenase from Pseudomonas sp. are purchased from Sigma-Aldrich Chemie GmbH (Steinheim, Germany)

Characterization methods

^{1}H and ^{13}C NMR spectra for all compounds were measured using the Bruker Avance 250 MHz and 300 MHz. UV-Vis absorption spectra of were recorded on a Perkin Elmer Lambda 25 UV-vis spectrometer and Thermo Scientific NanoDrop 8000 spectrophotometer. Solid-state diffuse reflectance UV-Vis absorption and fluorescence spectra were recorded on a Perkin Elmer Lambda 100 spectrophotometer and J&M TIDAS spectrofluorometer at ambient temperature,

respectively. Nitrogen sorption was measured using a Micromeritics Tristar II Plus with samples degassed for 12 hours at 120 °C under vacuum prior to analysis. Morphology of nanoparticles were examined with a Gemini 1530 (Carl Zeiss AG, Oberkochem, Germany) scanning electron microscope (SEM) operating at 0.35 kV and a Jeol 1400 (Jeol Ltd, Tokyo, Japan) transmission electron microscope (TEM) operating at an accelerating voltage of 120 kV. SEM and TEM samples of nanoparticles were prepared by casting the diluted dispersion on silicon wafers and carbon layer-coated copper grids, respectively. Cyclic voltammetry measurements were carried out on a Metrohm Autolab PGSTAT204 potentiostat/galvanostat with a three-electrode-cell system: glassy carbon electrode as the working electrode, $Hg/HgCl_2$ electrode as the reference electrode, platinum wire as the counter electrode, and Bu_4NPF_6 (0.1 M in acetonitrile) as supporting electrolyte with a scan rate of 100 mV s^{-1} in the range of -2 eV to 2 eV. All DFT calculations were carried out with the Gaussian 09W package. The structures were optimized at the B3LYP level of theory,[1] with the basis set of 6-31G*.[2] Thermogravimetric analysis (TGA) was conducted in an air atmosphere with temperature increasing from room temperature to 800 °C at a rate of 10 K min^{-1}. FT-IR measurements were conducted with a Varian 1000 FT-IR spectrometer. EPR (Electron Paramagnetic Resonance) was measured on a Magnettech Miniscope MS200 spectrometer at room temperature, microwave frequency: 9.391 GHz, microwave power: 10 mW, modulation amplitude: 9.8 G, field modulation: 0.2 mT at 100 kHz, scan time: 60 s.

Synthesis of $BTPh_2$-bridged organosilica monomer

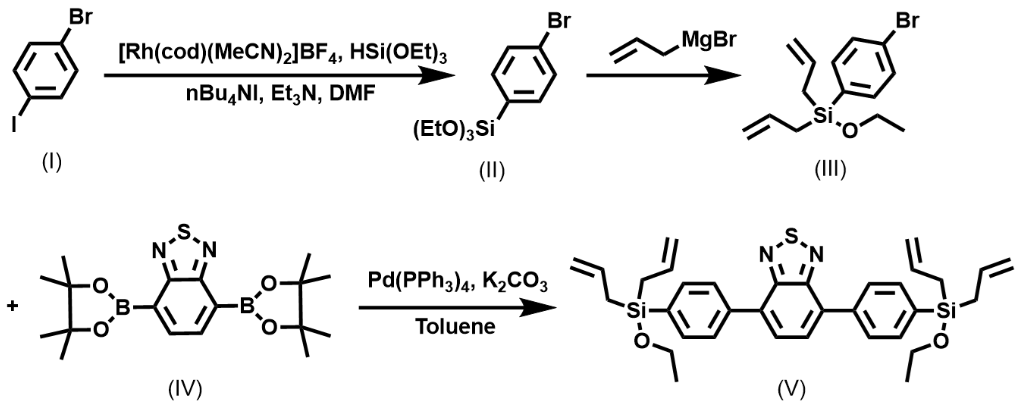

Scheme S1 Synthetic route of $BTPh_2$-bridged organosilica monomer.

4-Bromophenyltriethoxysilane[3] (II): 1-brom-4-iodobenzene (563 mg, 2 mmol), triethoxysilane (0.73 mL, 4 mmol), triethylamine (0.83 mL, 12 mmol) and bis(acetonitrile)(1,5-cyclooctadiene)rhodium(I)tetrafluoroborate (22 mg, 0.06 mmol) were dissolved in dry DMF (8 mL) and added in 25 mL Schlenk tube. Then, the Schlenk tube was degas in liquid nitrogen temperature and backfilled with argon 3 times. After degassing process, the reactive mixture was heated at 80 °C for 2 hours. While cooled down in room temperature, the mixture was diluted with 100mL diethyl ether and washed with NH_4Cl saturated solution 3 times. A crude product was obtained after drying over anhydrous $MgSO_4$ and purified by chromatography on silica with petroleum ether/dichloromethane (1/1) as the elution to afford the pure product as clear light yellow oil.

^{1}H NMR (300 MHz, $CDCl_3$) δ 7.52 (d, *J*=8.4 Hz, 2H), 7.43 (d, *J*=8.4 Hz, 2H), 5.79 (ddt, *J*=15.7 Hz, 9.7 Hz, 7.8 Hz, 2H), 4.95 (ddt, *J*=15.7 Hz, 1.6 Hz, 1.4 Hz, 2H), 4.92 (ddt, *J*=9.7 Hz, 1.6 Hz, 1.1 Hz, 2H), 3.76 (q, *J*=6.8 Hz, 2H), 1.91 (ddd, *J*=7.8 Hz, 1.4 Hz, 1.1 Hz, 4H)1.21 (t, *J*=6.8 Hz, 3H);

^{13}C NMR (75 MHz, $CDCl_3$) δ 135.50, 133.88, 132.63, 130.90, 124.68, 114.96, 59.27, 21.08, 18.32.

1-Bromo-4-(diallylethoxysilyl)benzene[3] (III): 4-bromophenyltriethoxysilane (474 mg, 1.49 mmol) was added allylmagnesium bromide (5.94 mL, 1 M in diethyl ether, 5.94 mmol) in diethyl ether with argon atmosphere. The reaction mixture was stirred at room temperature for 10 hours and quenched with 10% HCl. It was then diluted with diethyl ether and the organic layer was washed with saturated $NaHCO_3$ solution and brine, dried over anhydrous $MgSO_4$, and evaporated under reduced pressure. The crude mixture was purified by chromatography on silica with to give the pure product as yellow oil.

^{1}H NMR (300 MHz, $CDCl_3$) δ7.52 (d, *J*=8.4 Hz, 2H), 7.48 (d, *J*=8.4 Hz, 2H), 5.81 (ddt, *J*=15.7 Hz, 9.7 Hz, 7.8 Hz, 2H), 4.95 (ddt, *J*=15.7 Hz, 1.6 Hz, 1.4 Hz, 2H), 4.92 (ddt, *J*=9.7 Hz, 1.6 Hz, 1.1 Hz, 2H), 3.76 (q, *J*=6.8Hz, 2H), 1.91 (ddd, *J*=7.8 Hz, 1.4 Hz, 1.1Hz, 4H),1.21 (t,*J*=6.8Hz, 3H);

^{13}C NMR (75 MHz, $CDCl_3$) δ 135.50, 133.88, 132.63, 130.90, 124.68, 114.96, 59.27, 21.08, 18.32.

4,7-bis(4-allylethoxysilylphenyl)-2,1,3-benzothiadiazole (V): To a mixture of 4-(diallylethoxysilyl)bromobenzene (164.3 mg, 0.60 mmol), $Pd(PPh_3)_4$ (16.9 mg, 0.015 mmol), K_2CO_3 (101 mg, 0.73 mmol), and 2,1,3-benzothiadiazole-4,7-bis(boronic acid pinacol ester) (97.6 mg, 0.25 mmol) was added toluene (5 mL). The reaction mixture was degassing in liquid nitrogen temperature and backfilled with argon 3 times. After degassing process, the reactive mixture was heated at 80 °C for 24 hours. Then the reaction mixture was diluted with diethyl ether, which was filtered through a Celite plug, and the filter cake was rinsed with diethyl ether. The combined filtrates were concentrated under reduced pressure. The residue was chromatographed on silica gel with petroleum ether/ethyl acetate (20/1) to give a sticky yellow oil.

^1H NMR (300 MHz, $CDCl_3$) δ 7.92 (d, J = 7.7 Hz, 2H), 7.77-7.66 (m, 2H), 7.50 (s, 1H), 5.79 (dq, J = 18.1, 9.6 Hz, 2H), 5.00-4.81 (m, 4H), 3.82-3.66 (m, 2H), 1.90 (dd, J = 20.1, 8.0 Hz, 4H), 1.17 (q, J = 6.7 Hz, 3H).

^{13}C NMR (75 MHz, $CDCl_3$) δ 154.04, 134.38, 133.22, 133.11, 129.27, 128.54, 128.32, 114.93, 59.42, 21.31, 18.46.

Preparation of NP-C and NP-CS

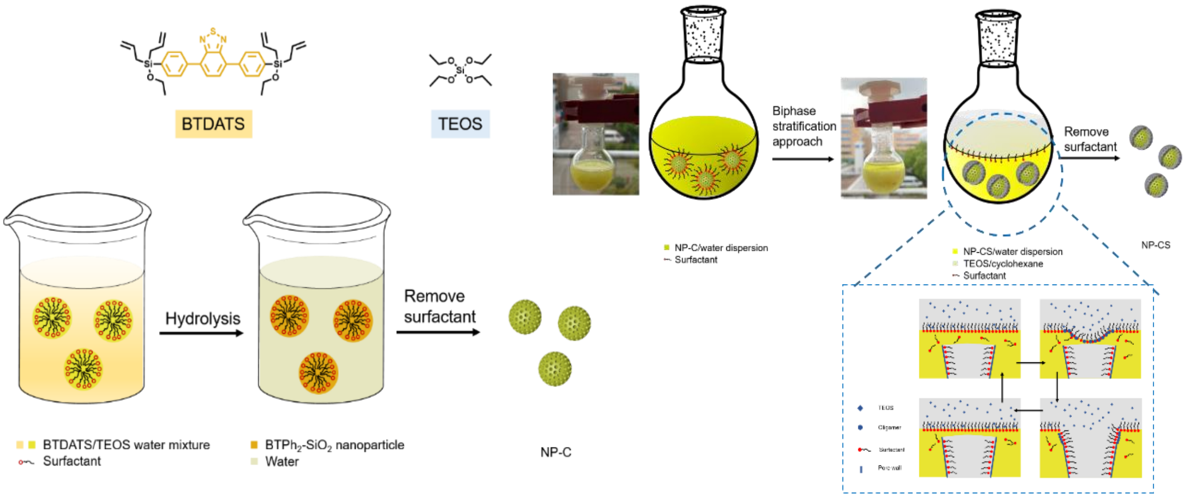

Scheme S2 Synthetic route for photocatalytic core nanoparticle (NP-C) and photocatalytic core-shell nanoparticle (NP-CS).

NP-C: A mixture of CTAB (25 mg), distilled water (12 mL), and sodium hydroxide (87.5 μL, 2 M) was stirred at 80 °C for 50 minutes at 700 rpm in a 25 mL round bottom flask. Then, TEOS (0.1 mL, 0.45 mmol) was added along with 4,7-bis(4-allylethoxysilylphenyl)-2,1,3-benzothiadiazole (26.7 mg in 1 mL of dry THF). The mixture was treated ultrasonically for 20 min to disperse the hydrophobic BTBADS in the aqueous solution, and the condensation process was carried out for 12 hours. Afterwards, the solution was cooled to room temperature with stirring. Fractions were gathered in propylene tubes and collected by centrifugation.[4]

NP-CS: 60 mg NP-C, 2.4 ml of (25 wt %) CTAC solution and 18mg of TEA were added to 3.6 ml of water and stirred gently at 60 °C for 1 h in a 10-mL round bottom flask, then 2 mL of (20 v/v %) TEOS in cyclohexane was carefully added to the water-CTAC-TEA solution and kept at 60 °C in an oil bath under a magnetic stirring. The stirring rate was set to be 150 rpm. The reaction was then kept at a constant temperature with continuous stirring for 16 h to obtain the products. The products were collected by centrifugation and washed for several times with ethanol to remove the residual reactants. Then, the collected products were extracted with a 0.6 wt % ammonium nitrate (NH_4NO_3) ethanol solution at 60.0 °C for 6 h twice to remove the template and dried in vacuum.[5]

Photocatalytic oxidation of NADH by NP-C and NP-CS

An aqueous dispersion (milliQ water, D_2O for NMR experiments) of NP-C and NP-CS (1 $mg{\cdot}mL^{-1}$) and NADH (10 $mmol{\cdot}L^{-1}$) were added to a 1 mL glass vial. The reaction mixture was stirred at 25 °C, saturated with O_2 or put in the air, irradiated with a blue-LED (460 nm, 0.10 W cm^{-2}) for 1 hour. At different time intervals, aliquots of the sample were collected and analyzed by solution ^1H NMR (250 MHz) and UV/Vis absorption spectroscopy. The changes of absorption of NADH at 340 nm were monitored over different time intervals respectively.

NAD^+/NADH interconversion under LED irradiation

With glycerol dehydrogenase: The reaction mixture was composed of NP-C (1 $mg{\cdot}mL^{-1}$) and NP-CS (3 $mg{\cdot}mL^{-1}$), glycerol dehydrogenase lyophilized powder (100 $\mu g{\cdot}mL^{-1}$), $(NH_4)_2SO_4$ (40 $mmol{\cdot}L^{-1}$) and NAD^+ (20 $mmol{\cdot}L^{-1}$) in Na_2CO_3/$NaHCO_3$ buffer (100 $mmol{\cdot}L^{-1}$, pH 9.5). To initiate the enzyme reaction, glycerol (0.5 $mmol{\cdot}L^{-1}$) was added to the mixture. The progress of the reaction was followed by the corresponding changes in the absorbance intensity of NADH at 340 nm. Absorbance data was collected every 30 minutes. Each cycle, dark and light,

was monitored for 30 minutes. For the light irradiation cycle, blue LED (460 nm, 0.10 W cm^{-2}) was used. Dark-Light cycles were repeated for ten times.

With glucose dehydrogenase: In 1 mL glass vial, NAD^+ (5 mmol·L^{-1}), NP-C (0.8 mg·mL^{-1}) and NP-CS (2.4 mg·mL^{-1}), glucose dehydrogenase (80 μg·mL^{-1}), were added in 0.6 mL phosphate buffer (50 mmol·L^{-1}, pH 7.4). To start reaction, glucose (1 mmol·L^{-1}) was added to the mixture and kept it under dark condition. Changes in absorbance at 340 nm were measured after 30 min. Then, blue LED (460 nm, 0.10 W cm^{-2}) was irradiated to the vial to activate NP-C and NP-CS for 30 min and the absorbance was measured. We repeated these procedures for ten times to obtain ten cycles.

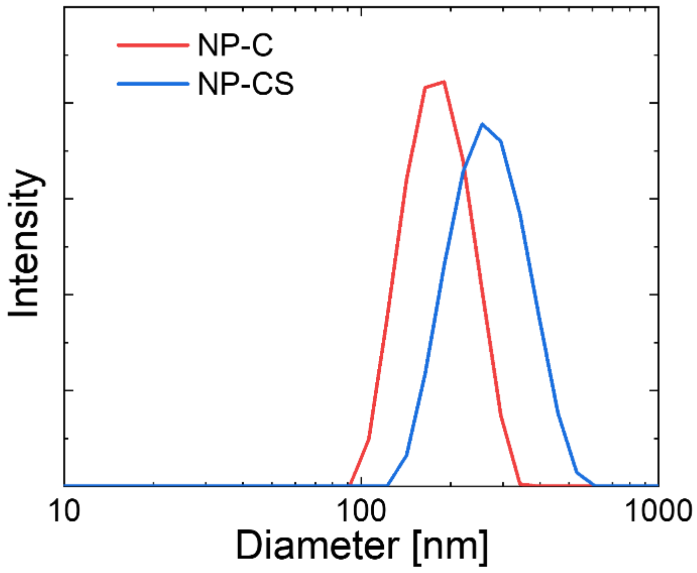

Figure S1. Dynamic light scattering (DLS) spectra of NP-C and NP-CS.

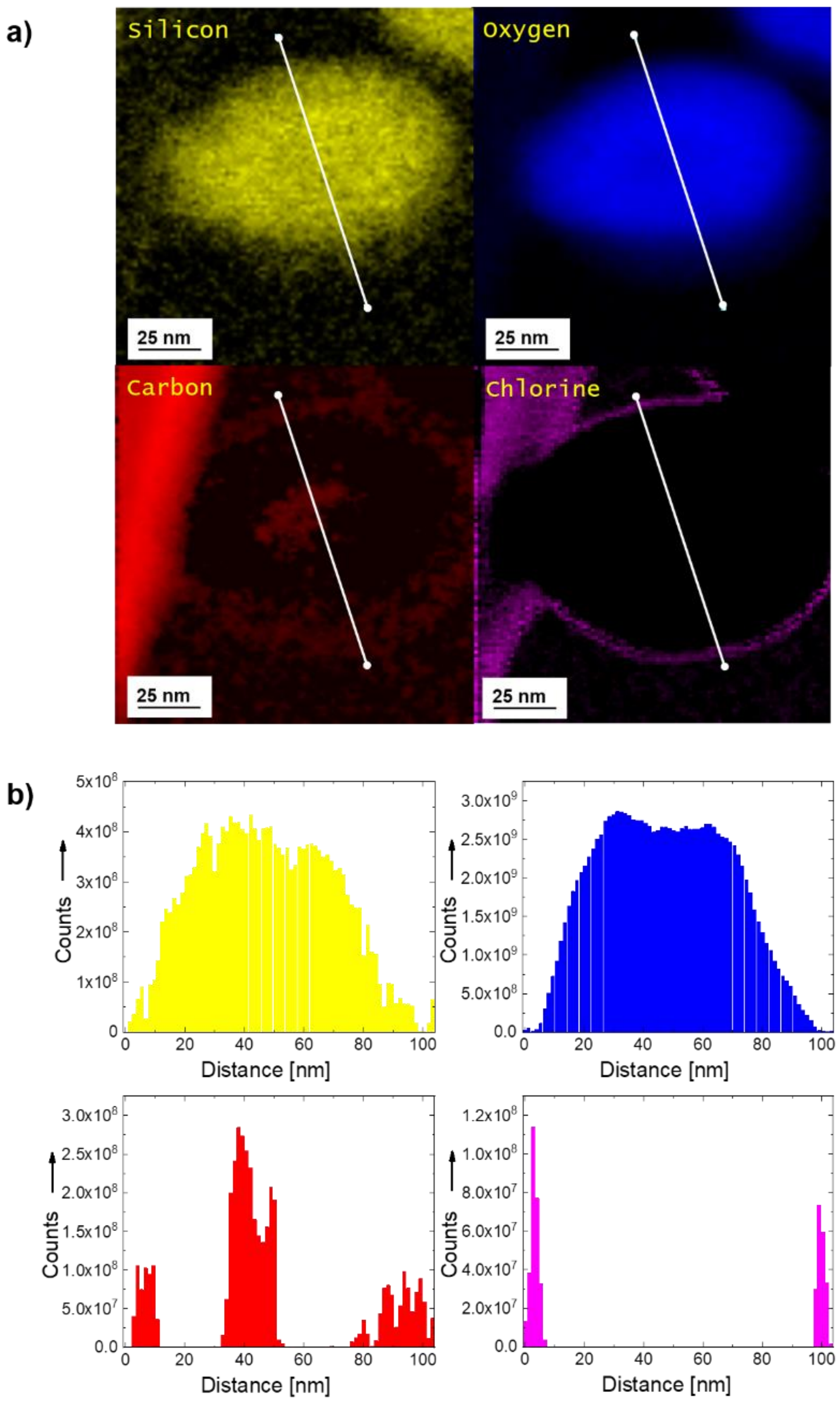

Figure S2. a) Elemental mapping and b) cross section elemental distribution of NP-CS.

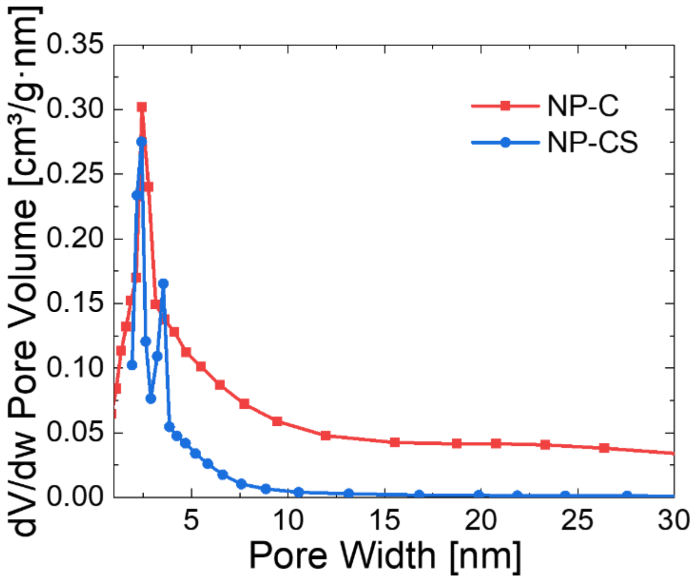

Figure S3. BJH pore size distributions of NP-C and NP-CS.

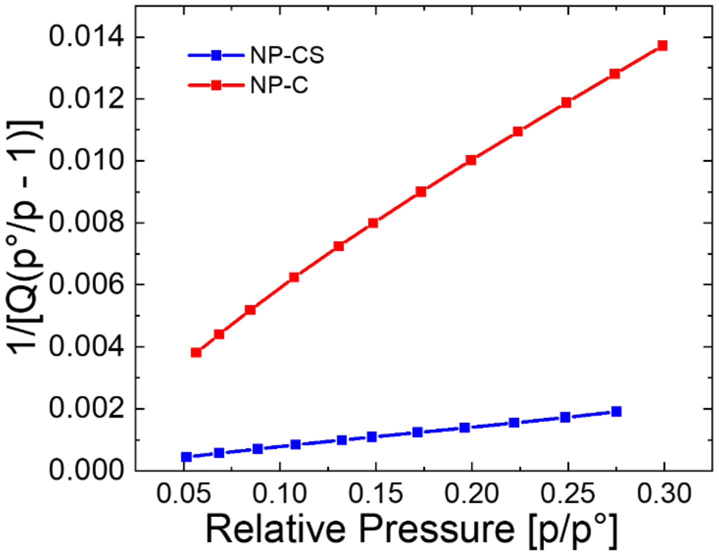

Figure S4. BET surface area plot of NP-C and NP-CS.

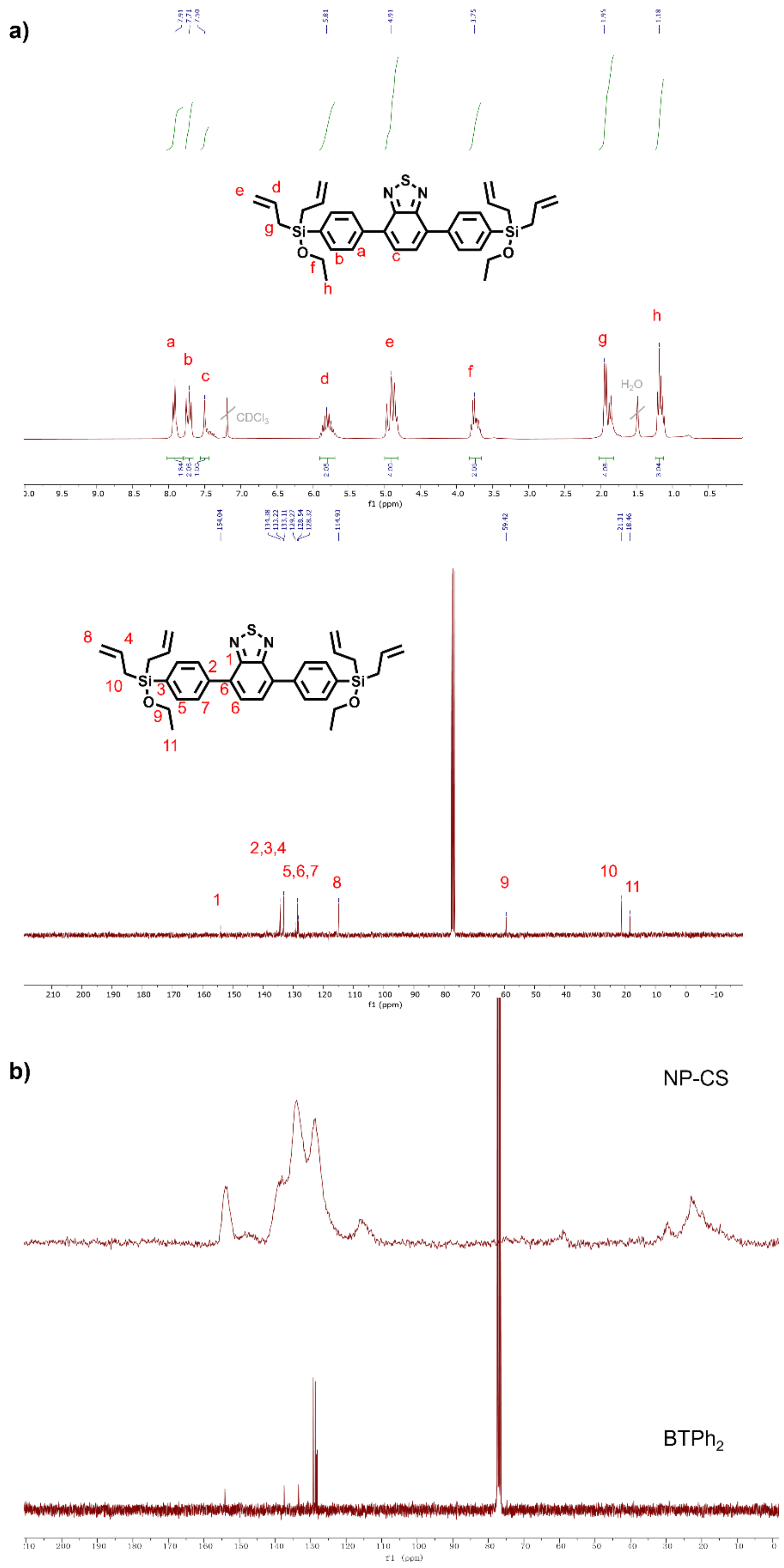

Figure S5. a) ^{1}H and ^{13}C NMR spectra of monomer 4,7-bis(4-allylethoxysilylphenyl)-2,1,3-benzothiadiazole; b) ^{13}C CP-MAS NMR spectrum of NP-CS and ^{13}C NMR ($CDCl_3$, 75 MHz) spectrum of diphenylbenzothiadiazole ($BTPh_2$).

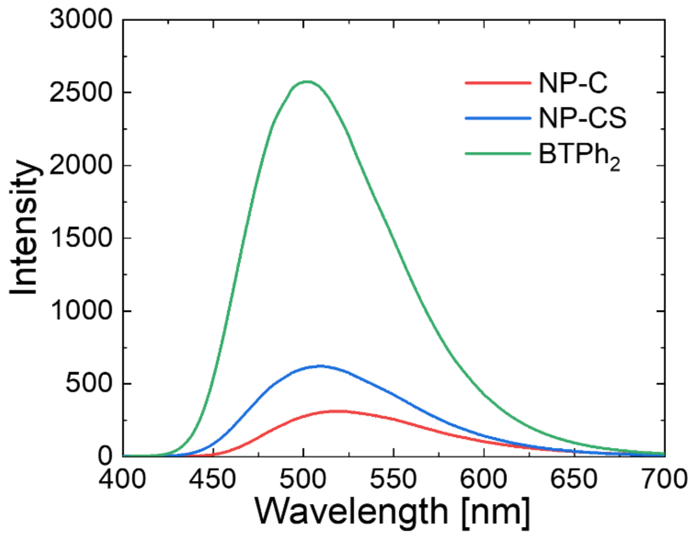

Figure S6. Comparison of the photoluminescence of $BTPh_2$, NP-C and NP-CS.

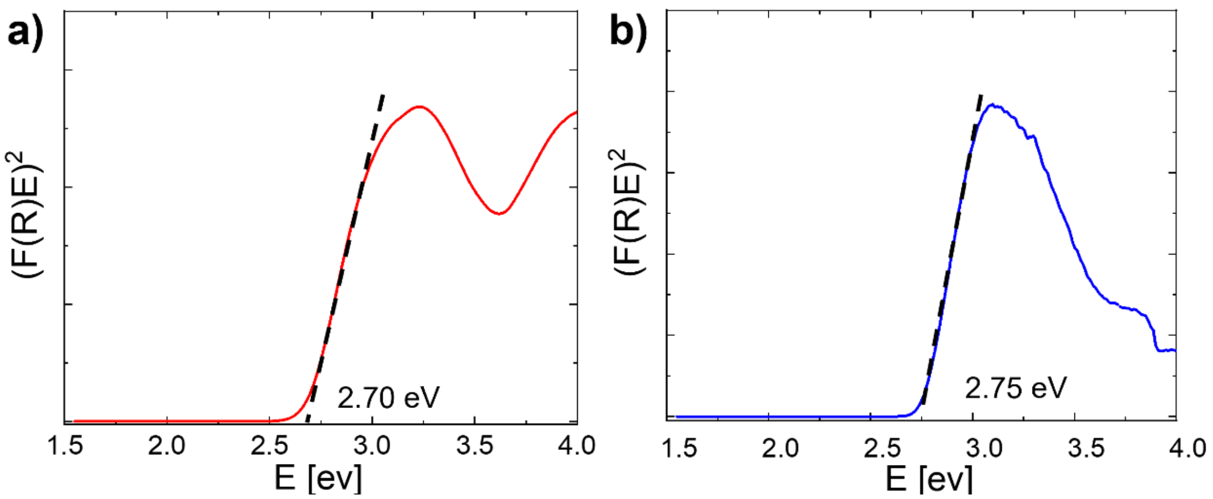

Figure S7. Kubelka-Munk transformed UV/Vis reflectance spectra of a) NP-C and b) NP-CS.

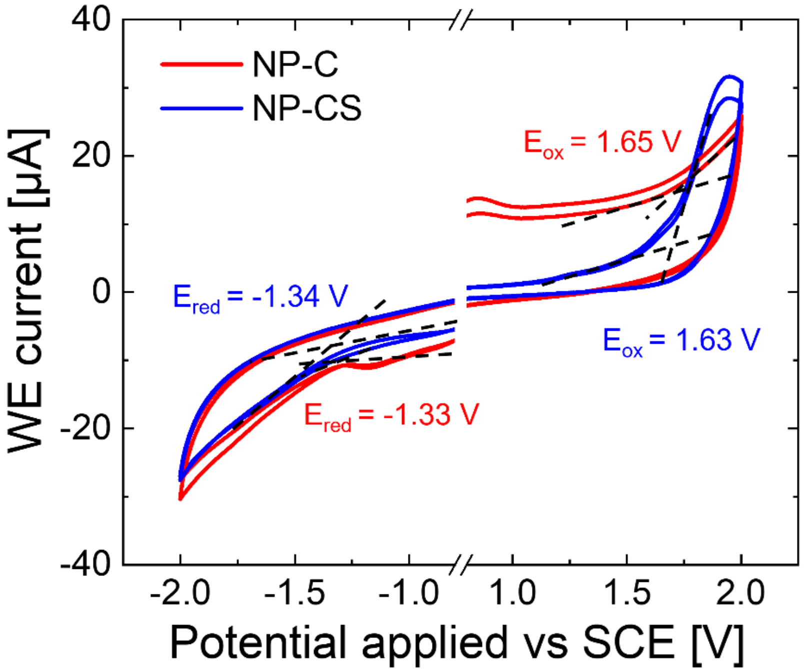

Figure S8. Cyclic voltammetry of photocatalytic nanoparticles.

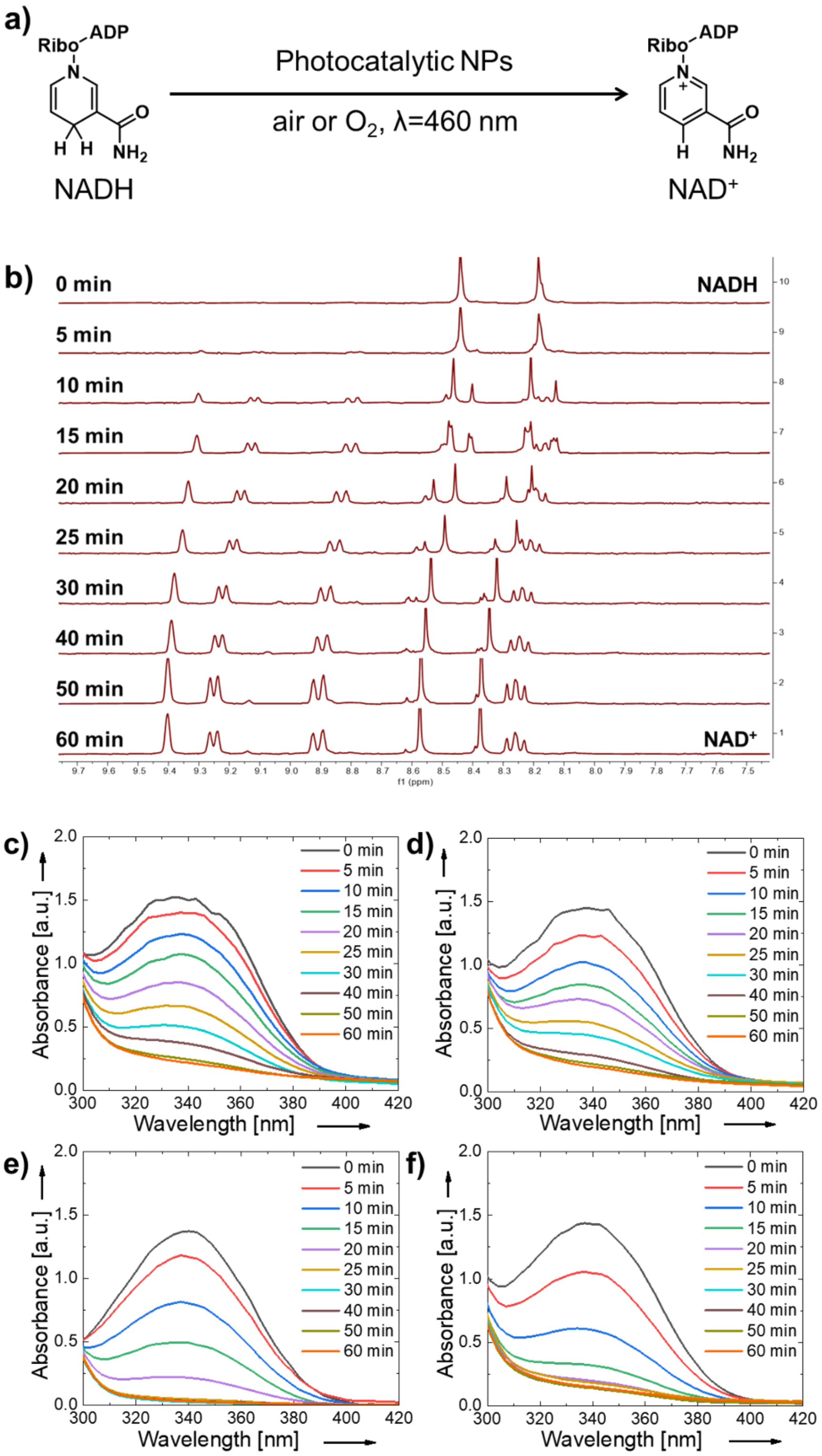

Figure S9. NADH oxidation with NP-C and NP-CS: a) equation; b) ^{1}H NMR spectra of NADH to NAD^{+} over time containing NP-CS in air and absorbance spectra of 1 hour NADH oxidation containg c) NP-CS with air, d) NP-CS with O_2, e) NP-C with air and f) NP-C with O_2.

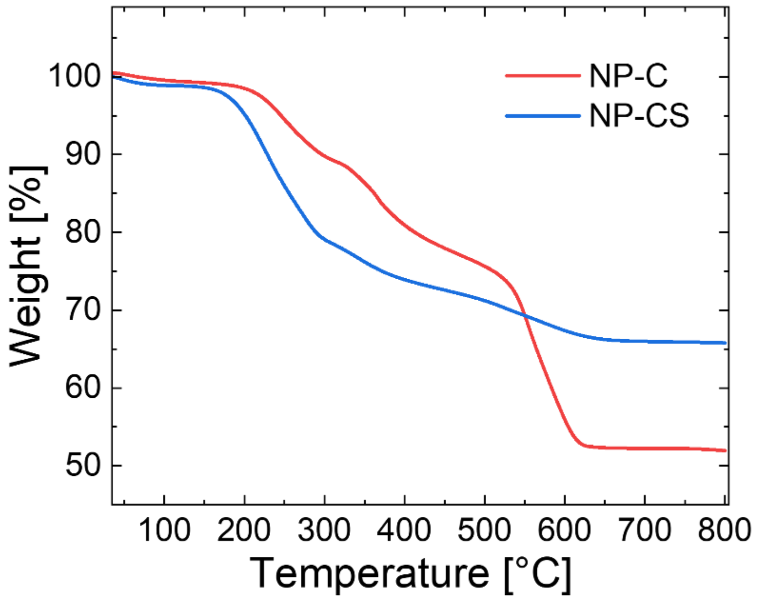

Figure S10. Thermogravimetric analysis (TGA) of NP-C and NP-CS under air with temperature increasing from room temperature to 800°C at a rate of 10 K/min. The decrease of the NP-CS curve at 200°C is attributed to the surfactant.

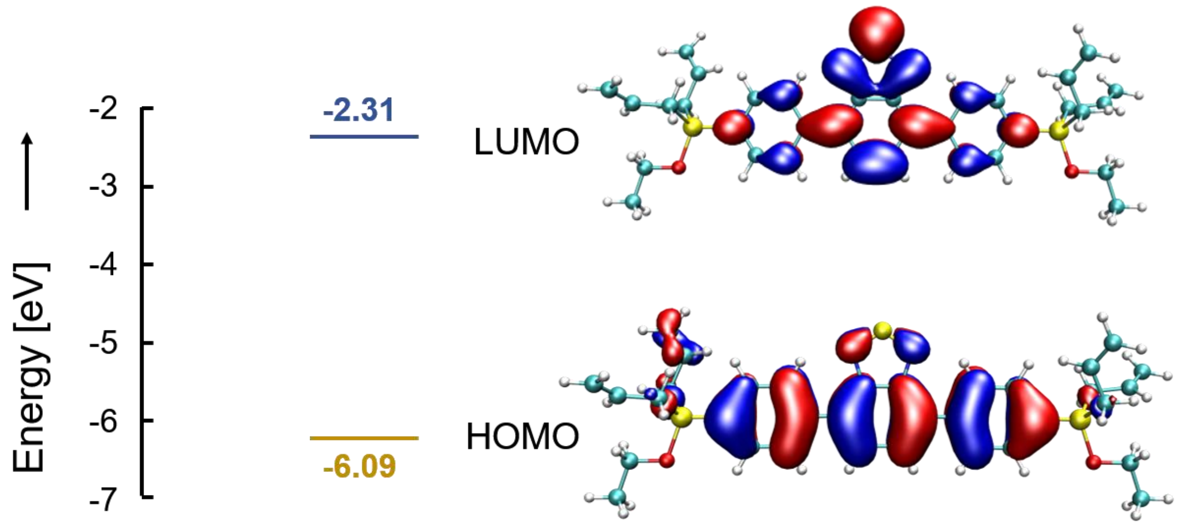

Figure S11. DFT Calculations on the B3LYP/6-31G(d) level for monomer 4,7-bis(4-allylethoxysilylphenyl)-2,1,3-benzothiadiazole.

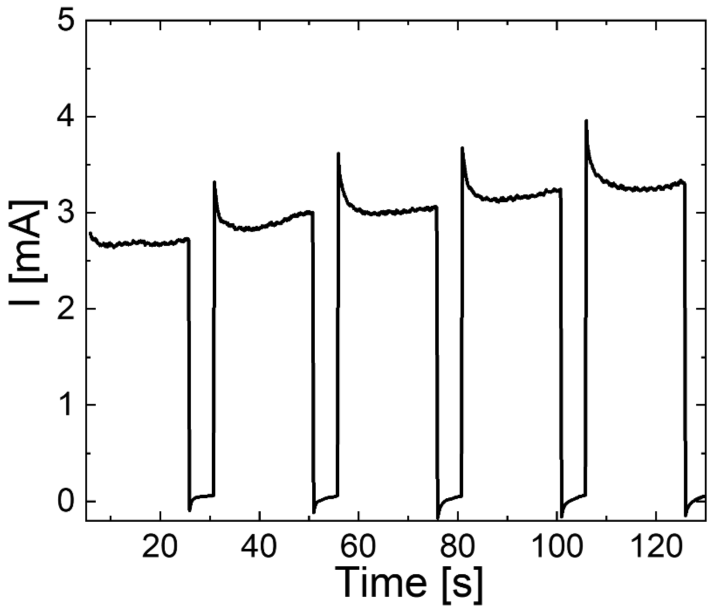

Figure S12. Photocurrent responses upon switching the light (blue LED, λ=460 nm) on and off (on: 25 s, off: 5 s).

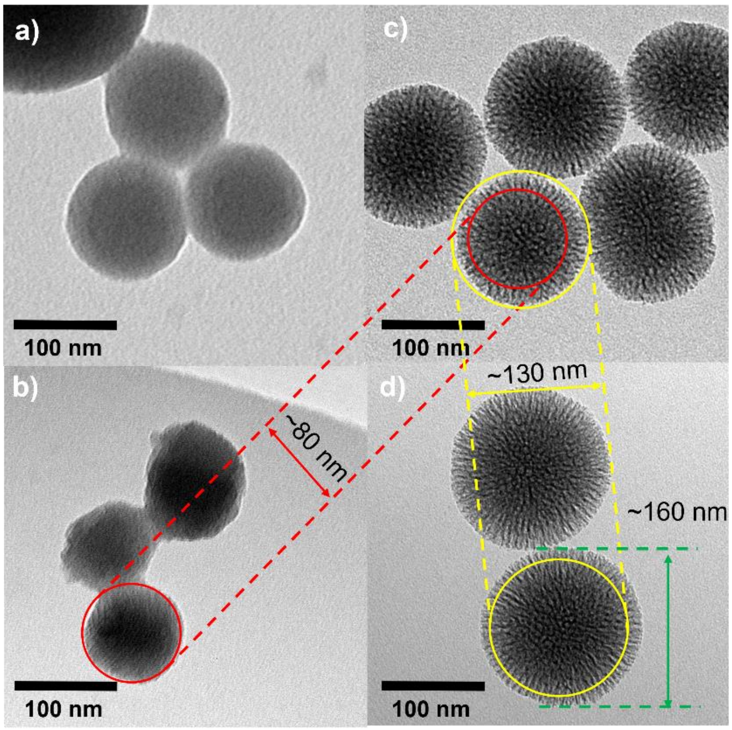

Figure S13. TEM images of NP-C and NP-CS with different shell growth method and time: a) NP-C with a non-porous shell by Stöber process; b) NP-C (no shell), c) NP-CS (with shell thickness ca. about 25 nm) and d) NP-CS (with shell thickness of ca. 40 nm).

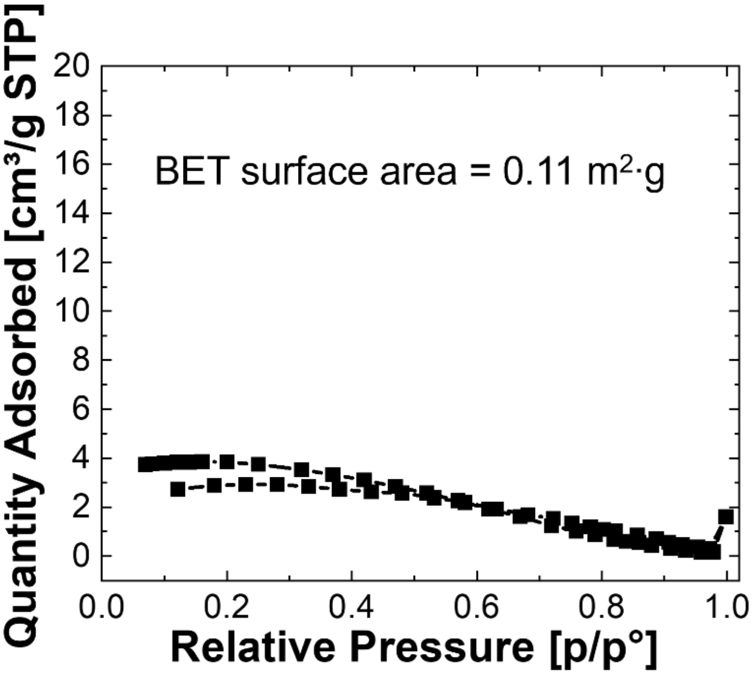

Figure S14. N_2 gas sorption isotherms of NP-C with a non-porous shell.

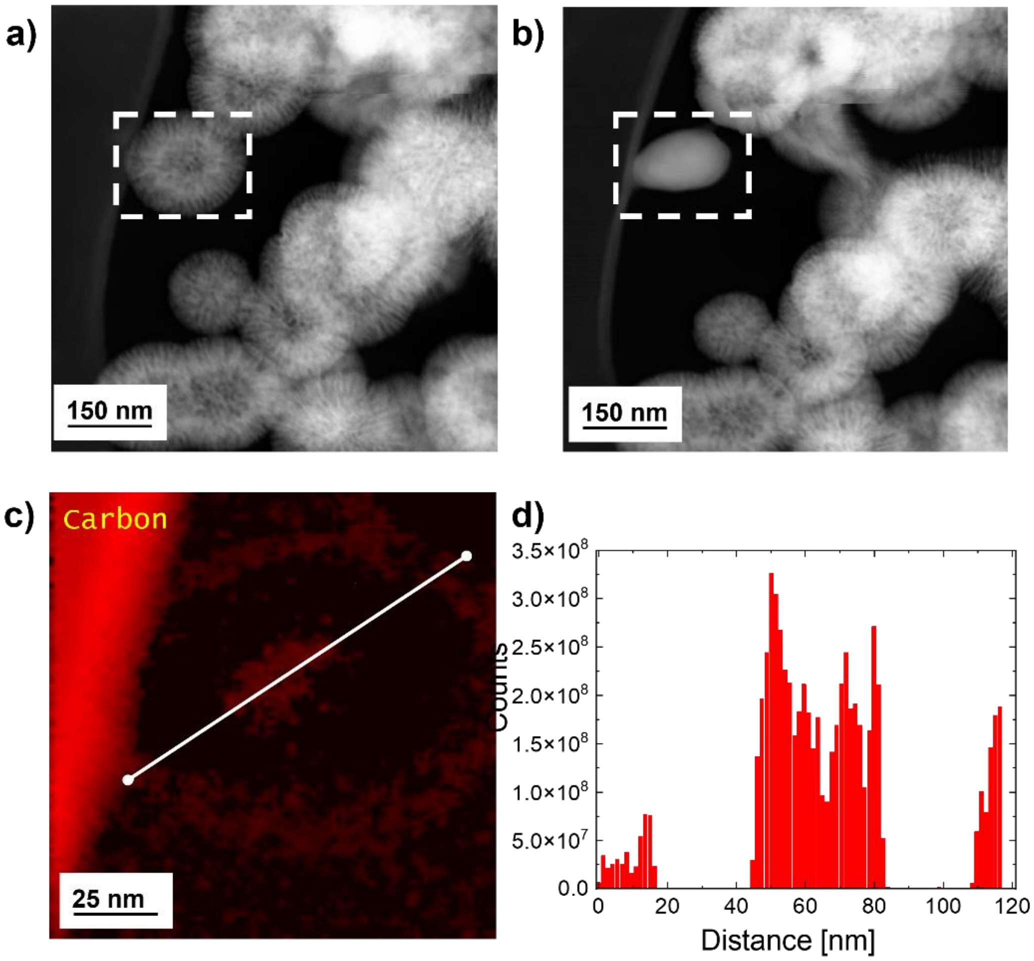

Figure S15. Annular dark-field (ADF) image of NP-CS a) before and b) after EELS measurement; c) Elemental mapping and d) cross section elemental distribution of NP-CS from major axis of ellipse. **Note:** Our finding is that electron beam significantly influenced the morphology of the sample during the EELS measurement. The shape of the nanoparticle was visiblly deformed into an ellipse.

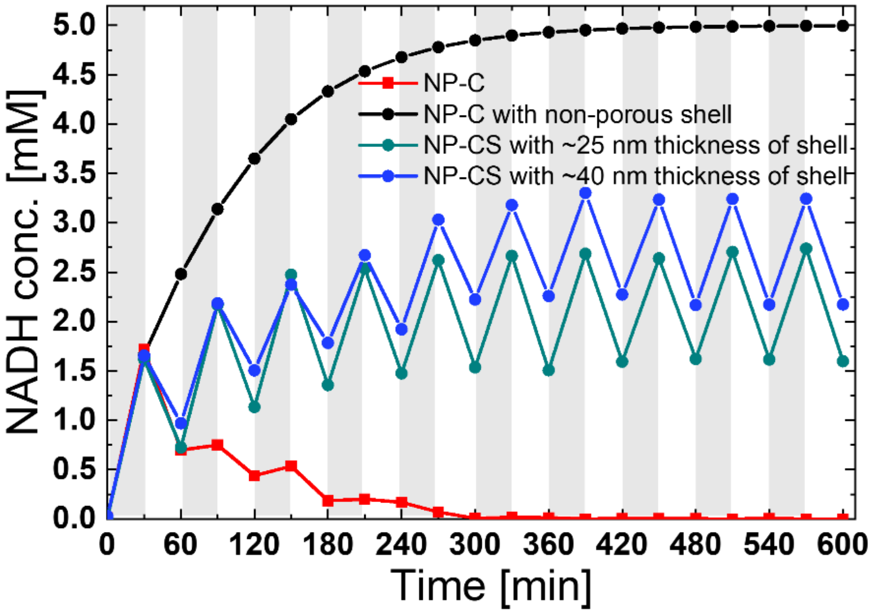

Figure S16. Control experiments of NP-C, NP-C with a non-porous shell, NP-CS with shell thickness of ca. 25 nm and NP-CS w with shell thickness of ca. 40 nm conbining with glucose dehydronase.

References

1. Patil, V. S.; Padalkar, V. S.; Tathe, A. B.; Gupta, V. D.; Sekar, N., Synthesis, Photo-physical and DFT Studies of ESIPT Inspired Novel 2-(2′,4′-Dihydroxyphenyl) Benzimidazole, Benzoxazole and Benzothiazole. *J. Fluoresc.* **2013,** *23* (5), 1019-1029.
2. McLean, A. D.; Chandler, G. S., Contracted Gaussian basis sets for molecular calculations. I. Second row atoms, Z=11–18. *J. Chem. Phys.* **1980,** *72* (10), 5639-5648.
3. Maegawa, Y.; Nagano, T.; Yabuno, T.; Nakagawa, H.; Shimada, T., Preparation of functionalized aryl(diallyl)ethoxysilanes and their palladium-catalyzed coupling reactions giving sol–gel precursors. *Tetrahedron* **2007,** *63* (46), 11467-11474.
4. Croissant, J. G.; Qi, C.; Mongin, O.; Hugues, V.; Blanchard-Desce, M.; Raehm, L.; Cattoën, X.; Wong Chi Man, M.; Maynadier, M.; Gary-Bobo, M.; Garcia, M.; Zink, J. I.; Durand, J. O., Disulfide-gated mesoporous silica nanoparticles designed for two-photon-triggered drug release and imaging. *J. Mater. Chem. B* **2015,** *3* (31), 6456-6461.
5. Shen, D.; Yang, J.; Li, X.; Zhou, L.; Zhang, R.; Li, W.; Chen, L.; Wang, R.; Zhang, F.; Zhao, D., Biphase Stratification Approach to Three-Dimensional Dendritic Biodegradable Mesoporous Silica Nanospheres. *Nano Letters* **2014,** *14* (2), 923-932.
